# Biomimetic Coverage Optimization in WSNs via an Enhanced Spider-Wasp Optimizer with Adaptive Learning and Dual-Median-Point Guidance

**DOI:** 10.3390/biomimetics11100760

**Published:** 2026-10-21

**Authors:** Trong-The Nguyen, Longcun Wang, Thi-Kien Dao

**Affiliations:** 1 School of Electronic Engineering, Fuzhou Institute of Technology, Fuzhou 350506, China; thent@uit.edu.vn (T.-T.N.); wanglc@gmiot.com (L.W.); 2 Multimedia Communications Laboratory, University of Information Technology, VNU-Ho Chi Minh City, Ho Chi Minh City 70000, Vietnam

**Keywords:** wireless sensor networks, coverage optimization, spider-wasp optimizer, enhanced spider-wasp optimizer, learning strategy, dual-median-point guidance

## Abstract

Coverage optimization in wireless sensor networks (WSNs) is a non-convex deployment problem on which population-based optimizers often converge prematurely and lose diversity. This paper proposes the Enhanced Spider-Wasp Optimizer (ESWO), an extension of the Spider-Wasp Optimizer (SWO) conceptually inspired by the foraging and nesting behavior of Pompilidae wasps. ESWO integrates (i) an Adaptive Learning Strategy (ALS) that uses four types of positional gaps among individuals to sustain diversity, (ii) a dual-median-point guidance (DMPG) mechanism that steers exploitation towards the midpoint of two elite solutions, and (iii) a position-update rule with nest-overlap avoidance. Coverage is evaluated on a discretized target grid with a Euclidean binary-disk sensing model and an energy-penalized fitness. In four scenarios (25–65 nodes; 50 × 50 to 165 × 165 m^2^), ESWO was compared with seven algorithms, including SWO. It achieved coverage rates of 81%, 88%, 92% and 85%, the highest in every scenario (tied with MGWO at 81% in the smallest). In the 100 × 100 m^2^ scenario it reached 85% coverage after 523 iterations, against 601–698 for the competitors, although final convergence iteration counts were not uniformly the lowest. Over 15 runs, ESWO obtained a mean coverage of 90.2% (SD 1.2), a 95% success rate and the lowest estimated energy consumption (175.3 J).

## 1. Introduction

Wireless sensor networks (WSNs) consist of a collection of spatially distributed autonomous sensors deployed to monitor and record the physical conditions of an environment [1] and to cooperatively transmit their data to a central location [2,3]. These networks play a crucial role in a wide range of applications, including environmental monitoring, precision agriculture, industrial automation, healthcare, and disaster management [4,5]. The proliferation of Internet of Things (IoT) applications has further amplified the importance of WSNs, making them fundamental components of modern smart systems and cyber-physical infrastructures [6].

Coverage optimization in WSNs is particularly critical in several real-world applications where comprehensive monitoring directly impacts system effectiveness and operational success. In environmental monitoring systems, such as tracking air and water pollution levels, forest fire detection, or wildlife habitat observation, optimal sensor placement ensures that critical areas are monitored without gaps, enabling timely detection of environmental changes and efficient resource utilization while minimizing the total number of deployed sensors [7]. Precision agriculture systems rely on WSNs to monitor soil moisture, temperature, nutrient levels, and crop health across vast agricultural fields; optimized coverage in these applications reduces the number of sensors required while ensuring uniform data collection across the entire farming area, leading to significant cost savings, improved crop yields, and efficient resource management [8]. Smart city infrastructures deploy extensive sensor networks for traffic management, air quality monitoring, waste management, and public safety applications; optimal coverage strategies in urban environments ensure that city areas are efficiently monitored while improving citizen services and minimizing deployment and maintenance costs [9]. Industrial surveillance and monitoring systems, particularly in large manufacturing facilities, oil refineries, or remote industrial sites, require strategically placed sensors for equipment health monitoring, safety compliance, environmental hazard detection, and intrusion prevention; coverage optimization in these critical settings is essential to avoid dangerous blind spots that might lead to safety hazards, equipment failures, or security breaches [10]. In each of these application domains, the inherent challenges of varying terrain characteristics, physical obstacles, limited energy resources, and harsh environmental conditions make sophisticated coverage optimization strategies absolutely essential for practical and cost-effective WSN deployment [11].

A fundamental challenge in effectively deploying WSNs lies in achieving optimal coverage, which ensures comprehensive monitoring of the target area while minimizing energy consumption to prolong the network’s operational lifetime [12]. Coverage optimization in WSNs is inherently a multi-objective problem that must balance several competing factors: maximizing area coverage, minimizing energy consumption, ensuring network connectivity, and maintaining fault tolerance [13]. This challenge is exacerbated by factors such as limited sensor resources, varying terrain characteristics, irregular deployment areas, and the presence of obstacles that can impede signal propagation and hinder sensor effectiveness [14]. Additionally, the finite battery life of sensor nodes and the often inaccessible nature of deployment environments make energy-efficient coverage strategies paramount for practical WSN implementations [15].

Traditional optimization methods often struggle to address the complexities of WSN deployment, particularly in navigating high-dimensional solution spaces and avoiding local optima [16]. Classical techniques such as linear programming, gradient descent, and greedy algorithms find it challenging to tackle the high-dimensional, non-convex, and combinatorial complex nature of the WSN coverage optimization problem [17]. These methods frequently become trapped in local optima and fail to identify globally optimal solutions, especially when dealing with irregular deployment areas, heterogeneous sensor capabilities, and dynamic environmental constraints [18]. Furthermore, the discrete nature of sensor placement decisions and the need to consider multiple conflicting objectives simultaneously render traditional optimization approaches inadequate for real-world WSN deployment scenarios [19].

Metaheuristic algorithms, inspired by natural processes, have emerged as promising alternatives for tackling these complex optimization problems [20]. These algorithms, which draw inspiration from biological evolution, swarm behavior, or physical phenomena [21], offer significant advantages in effectively exploring the search space, escaping local optima, and handling non-convexity [22]. Popular metaheuristic approaches for WSN optimization include Genetic Algorithms (GA) [23], Particle Swarm Optimization (PSO) [24], Ant Colony Optimization (ACO) [25], and more recent bio-inspired algorithms such as Gray Wolf Optimizer (GWO) [26] and Whale Optimization Algorithm (WOA) [27].

The Spider-Wasp Optimizer (SWO) [28], a recently developed metaheuristic algorithm inspired by the sophisticated foraging behavior of spider wasps, has demonstrated promising results in various optimization tasks due to its unique balance between exploration and exploitation phases. However, the original SWO has certain limitations that hinder its effectiveness in complex optimization landscapes like those encountered in WSN coverage optimization. These limitations include: (1) a tendency toward premature convergence when dealing with multi-modal optimization problems, (2) difficulty in maintaining population diversity throughout the optimization process, particularly in later iterations, and (3) insufficient exploitation capabilities when fine-tuning solutions near optimal regions [29]. These shortcomings can significantly impact the algorithm’s performance in finding high-quality solutions for WSN coverage optimization, where the solution space is highly complex and requires both robust exploration of diverse regions and intensive exploitation of promising areas [17]. To address these limitations and enhance the effectiveness of WSN coverage optimization, this paper introduces an Enhanced Spider-Wasp Optimizer (ESWO), which builds upon the strengths of the original SWO while systematically addressing its weaknesses through three key innovations. The proposed ESWO incorporates advanced strategies to improve both exploration and exploitation capabilities, leading to superior performance in complex optimization scenarios typical of WSN deployment problems. The principal contributions of this paper are:

- ESWO algorithm: An enhanced SWO variant that adapts a learning strategy [30,31] and a dual-median-point guidance [32] and integrates them, together with a nest-overlap-avoidance update, into the SWO framework to improve coverage quality in the tested WSN scenarios.
- Explicit biomimetic mapping: A conceptual mapping between Pompilidae wasp behavior and each ESWO computational mechanism, providing a transparent biological rationale (an analogy, not an empirical validation).
- WSN coverage application: Application to WSN coverage optimization with a composite fitness function jointly addressing coverage rate and energy-consumption constraints.
- Comprehensive empirical validation: Systematic comparison against seven state-of-the-art algorithms across four deployment scales, varying sensing radii, obstacle densities, and energy metrics, supported by multi-run statistical analysis.

The rest of the paper is structured as follows: Section 2 reviews related work on WSN coverage optimization and the original SWO. Section 3 details the proposed ESWO method. Section 4 presents simulation results and comparisons, and Section 5 concludes the paper with future directions.

## 2. Related Work

In the realm of WSNs, coverage optimization is a critical challenge that has garnered significant attention from researchers. This section reviews the existing literature on coverage optimization strategies, highlighting the various approaches that have been developed to enhance coverage efficiency while addressing the inherent complexities of WSNs. The discussion is organized into two main subsections: the coverage optimization problem in WSNs and the SWO, which is a specific algorithm used in this context.

### 2.1. WSN Coverage Optimization Problem

The problem of coverage optimization in WSNs has been extensively studied, with various approaches proposed to address its inherent challenges [17]. Maximizing coverage efficiency requires strategically positioning sensor nodes to minimize coverage gaps while considering energy constraints [33]. The problem is further complicated by obstacles and variations in terrain, which can impact signal propagation and sensor effectiveness. Finding the optimal balance between coverage and energy efficiency is a complex optimization problem that necessitates advanced algorithms capable of navigating intricate solution spaces. These approaches can be broadly categorized into three main groups: geometric approaches, computational approaches, and metaheuristic approaches.

*Geometric approaches,* such as Voronoi diagrams and grid-based methods [34], offer computationally efficient solutions for coverage assessment. However, these methods often simplify the problem by assuming ideal sensor deployment conditions and neglecting factors like obstacles and variations in terrain. Consequently, they may not provide accurate coverage estimates in real-world scenarios.

*Computational approaches,* including integer programming and linear programming [35], aim to find optimal sensor placements by formulating the coverage problem as a mathematical optimization model. While these methods can guarantee optimality under certain conditions, they often become computationally intractable for large-scale WSNs due to the combinatorial nature of the problem [36].

*Metaheuristic algorithms,* inspired by natural processes, have gained significant traction in recent years for addressing complex optimization problems, including WSN coverage optimization. These algorithms, such as GAs [23], PSO [24], ACO [25], and GWO [26], offer a balance between solution quality and computational efficiency. They explore the search space effectively, escape local optima, and can handle non-convexity, making them well-suited for WSN coverage optimization. Several studies have employed metaheuristic algorithms for coverage optimization in WSNs. For instance, [37] proposed a GA-based approach for maximizing coverage while minimizing energy consumption. Reference [38] utilized PSO to optimize sensor placement for target coverage. Reference [39] employed ACO to find optimal coverage paths for mobile sensor networks.

Recent metaheuristic algorithms have significantly advanced WSN coverage optimization through enhanced convergence characteristics and improved solution quality. Energy-aware approaches include the Aware Gray Wolf Optimizer (AGWO) [40], which integrates energy-efficient clustering with coverage optimization in software-defined WSNs, and Enhanced Particle Swarm Optimization (EPSO) [41], which incorporates adaptive velocity updates for improved coverage uniformity and energy efficiency. Advanced search-based algorithms have addressed complex deployment scenarios through the Adapted Transit Search Algorithm (ATSA) [42], featuring enhanced local search capabilities and improved escape mechanisms, and the Enhanced Archimedes Optimization Algorithm (EAOA) [43], which achieves superior convergence through adaptive parameter adjustment mechanisms.

Bio-inspired enhancements include the Improved Flower Pollination Algorithm (IFPA) [44], which balances global and local search phases for random deployment scenarios, and the Improved Honey Badger Algorithm (IHBA) [45], incorporating novel foraging behaviors for multi-objective coverage optimization. Multi-strategy approaches represent current trends, with the Competitive Learning Optimizer (CLOM) [46] handling conflicting objectives through competitive learning mechanisms, and the Multi-strategy Enhanced Gray Wolf (MGWO) [47] targeting obstacle-aware environments with multiple search strategies and barrier avoidance capabilities. These enhanced algorithms consistently outperform traditional methods in coverage ratio, energy efficiency, and convergence speed. While these metaheuristic approaches have shown promising results, they still face challenges in balancing exploration and exploitation, maintaining population diversity, and efficiently navigating complex solution spaces.

### 2.2. Spider-Wasp Optimizer (SWO)

The SWO is a novel nature-inspired metaheuristic algorithm introduced by Abdel-Basset et al. in 2023 [28]. This algorithm draws its inspiration from the unique hunting, nesting, and mating behaviors exhibited by female spider wasps (Pompilidae family), which are solitary predatory insects renowned for their sophisticated prey-capture strategies [29]. Unlike conventional optimization algorithms that rely on abstract mathematical formulations, SWO mimics the intelligent behavioral patterns observed in nature, making it particularly suitable for solving complex optimization problems in wireless sensor networks.

**Figure 1:**
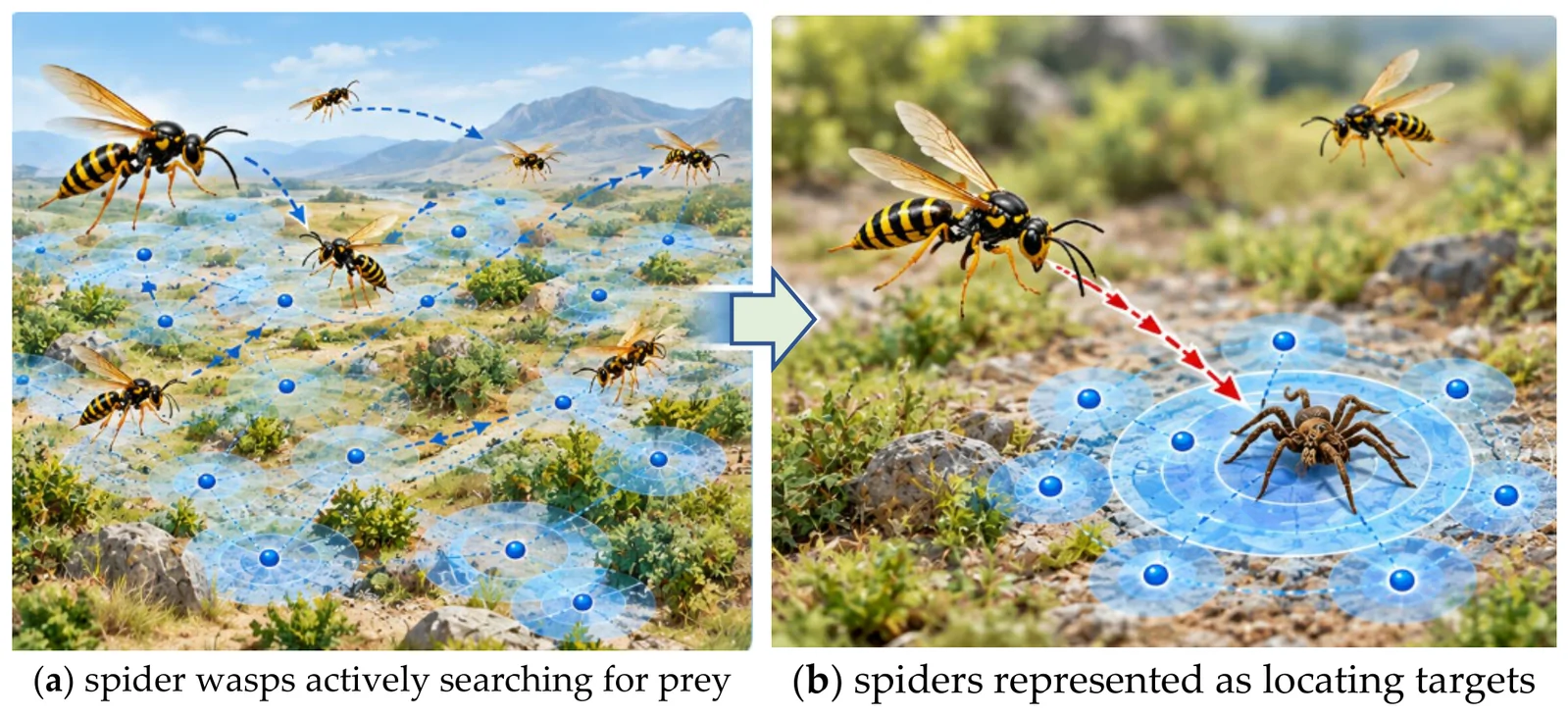
Hunting behavior mapping in the Spider-Wasp Optimizer.

Figure 1 illustrates how the characteristic hunting behavior of spider wasps maps to the two key phases of SWO algorithm, (a) spider wasps actively searching for prey, and (b) locating targets (represented as spiders) within the search space. During the exploration phase, the wasps’ broad search patterns mimic diverse spatial exploration. Upon detecting prey, their focused pursuit behavior corresponds to the exploitation phase, where sensor positions are refined around promising coverage regions. The biological model is inspired by the behavior of solitary predatory wasps (family Pompilidae), which locate, chase, and paralyze spiders as hosts for their larvae.

The SWO algorithm begins by initializing a population of potential solutions representing spider wasps in the search space. Let N be the population size and D be the dimensionality of the optimization problem. Each individual X in the population is represented as: Each individual X in the population can be represented as a position vector *X_i_* for a given individual index *i* (*i* = 1, 2, …, *N*):

(1) *X_i_* = [*x_i_*_1_, *x_i_*_2_, …, *x_iD_*],

where *x_i_*_,_*_j_* represents the position of the *i*-th individual in the *j*-th dimension. The initial positions are randomly generated within the defined search boundaries:

(2) *X_j_* = *L_j_* + *r* ⋅ (*H_j_* − *L_j_*),

where *L_j_* and *H_j_* are the lower and upper bounds for dimension *j*, respectively, and *r* is a random number uniformly distributed in [0,1]. Each spider’s fitness is assessed using a specified objective function, denoted as *f*(*x_i_*_,_*_j_*) = Objective Function. This fitness value indicates how near a given solution is to the optimal one. Depending on the nature of the problem, the algorithm seeks to either maximize or minimize this value. The hunting phase consists of multiple operations that simulate the natural prey-searching behavior of spider wasps. The spider searching operation simulates random search behavior when wasps explore their environment for potential prey:

(3) $$X_{i}^{t+1}=X_{i}^{t}+\mu_{1}\cdot (X_{a}^{t}-X_{b}^{t}),$$

where $X_{i}^{t+1}$ the current position of the *i*-th individual at iteration *t*, $X_{a}^{t}$ and $X_{b}^{t}$ are randomly selected positions from the current population, and μ_1_ is calculated as *r_n_* ⋅ *r*_1_, that is a motion control factor with *r*_1_, is a random value between 0 and 1, and *r_n_* is a normal distribution value in [0,1].

The following and escaping operation model the local search around a detected prey and an adaptive response to escape attempts:

(4) $$X_{i}^{t+1}=X_{c}^{t}+\mu_{2}\cdot (L+r_{2}\cdot (H-L)),$$

where $X_{c}^{t}$ represents a reference position in the search space, μ_2_ is set to B ⋅ cos(2πL), with B = 1/(1 + *e^l^*) and *l* is a random value in [−2,−1]. The movement of each wasp is influenced by the positions of the spiders and the best solution found. When actively pursuing detected prey, the chase operation updates positions using the update equation for the position of wasp:

(5) $$X_{i}^{t+1}=X_{i}^{t}+C\cdot (2\cdot r_{3}\cdot (X_{\mathrm{best}}^{t}-X_{i}^{t})),$$

where *C* is a linearly decreasing control parameter calculated as *C* = 2 − 2 · *t*/*tmax*, $X_{\mathrm{best}}^{t}$ represents the best solution found so far, and r_3_ is a random number between 0 and 1. When prey attempts to escape, the position adjustment follows:

(6) $$X_{i}^{t+1}=X_{i}^{t}\cdot v_{c},$$

where *v_c_* is an adaptive velocity vector that depends on the escape dynamics and distance parameters. The nesting phase simulates the optimization behavior where individuals refine their positions based on the best available solutions:

(7) $$X_{i}^{t+1}=X_{\mathrm{best}}^{t}+\cos(2\pi L)\cdot (X_{\mathrm{best}}^{t}-X_{i}^{t}),$$

where $X_{\mathrm{best}}^{t}$ is the global best position discovered and *l* is a random parameter that controls the intensity of local search around the optimal solution. To maintain an appropriate balance between exploration and exploitation, SWO employs a dynamic population reduction mechanism:

(8) *N^t^*^+1^ = *N_min_* + (*N^t^* − *N_min_*) ∗ *k*,

where *N_min_* is the minimum population size required to maintain diversity and *k* is a reduction factor that gradually decreases the population size to prevent premature convergence while ensuring computational efficiency. Algorithm 1 presents the pseudocode of the Spider-Wasp Optimizer (SWO).
**Algorithm 1**: Spider-Wasp Optimizer (SWO) Pseudocode
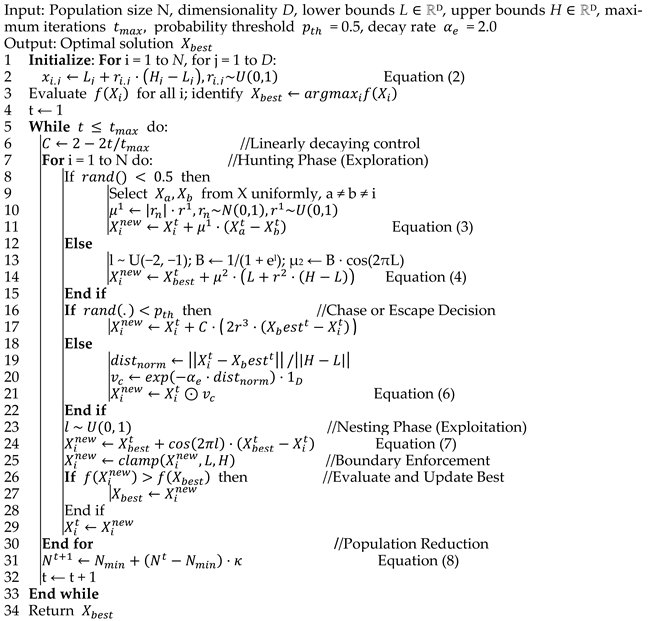


The SWO offers several distinctive advantages for wireless sensor network coverage optimization. The algorithm provides natural mechanisms for balancing global search with local refinement, crucial for avoiding local optima in complex WSN coverage landscapes. Its population reduction strategy allows adaptive computational resource management throughout the optimization process, improving convergence efficiency. The diverse behavioral operations enable SWO to effectively handle multi-modal optimization problems common in sensor placement scenarios. By leveraging bio-inspired parameter adaptation mechanisms, the algorithm reduces the need for extensive parameter tuning while maintaining optimization effectiveness.

## 3. The Enhanced Spider-Wasp Optimizer: Biological Inspiration and Algorithmic Design

### 3.1. Biological Inspiration and Motivation for ESWO Design

Pompilidae wasps are solitary predators that provision their nests with paralyzed spiders. Their foraging behavior consists of four phases that naturally reflect the exploration–exploitation balance in metaheuristic optimization, as illustrated in Figure 1.

Phase 1—Broad-area search: The wasp performs wide-ranging movements to explore a large territory, analogous to global exploration across the search space.

Phase 2—Targeted pursuit and paralysis: After detecting a spider, the wasp switches to focused pursuit and immobilizes the prey, corresponding to exploitation around promising solutions.

Phase 3—Adaptive escape response: When the spider attempts to escape, the wasp dynamically adjusts its trajectory to maintain contact, resembling adaptive local search for escaping local optima.

Phase 4—Nesting and provisioning: The immobilized spider is transported and precisely placed in the nest, analogous to fine-grained local exploitation and solution refinement.

This behavioral cycle provides a biomimetic basis for balancing exploration and exploitation through adaptive transitions between broad search, focused pursuit, adaptive response, and precise refinement.

**Figure 2:**
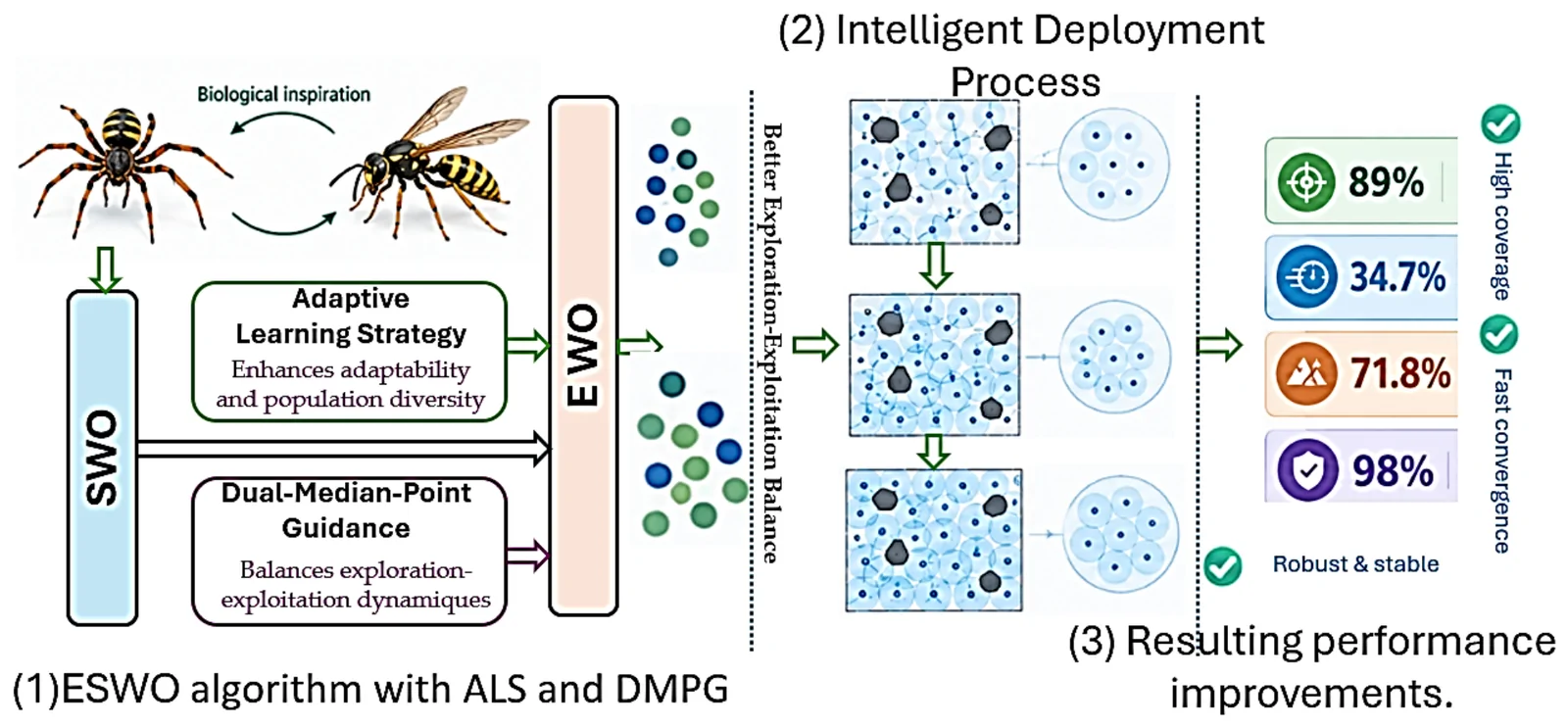
Framework of WSN deployment via the ESWO.

Although the baseline SWO [28] has shown competitive performance on benchmark functions and mobile robot path planning, its application to WSN coverage optimization faces three limitations: (1) premature convergence in multimodal landscapes, (2) loss of population diversity due to excessive attraction towards the global best, and (3) insufficient local refinement near promising deployment regions. These limitations can reduce coverage quality under practical energy and connectivity constraints [29]. To overcome these issues, the ESWO introduces three complementary mechanisms:

(i) Adaptive Learning Strategy (ALS): Enhances population diversity and global exploration by exploiting structured information gaps among individuals, corresponding to the broad-area search in Phase 1.
(ii) Dual-Median-Point Guidance (DMPG): Provides informative search directions using the median point of two elite solutions, strengthening exploitation of promising regions, corresponding to Phases 2 and 4.
(iii) Improved Position-Update Framework: Integrates ALS and DMPG with nest-overlap avoidance to improve the exploration–exploitation balance and escape local optima, reflecting the adaptive response in Phase 3. Together, these mechanisms enhance the diversity, convergence, robustness, and energy efficiency of SWO for WSN coverage optimization. Figure 2 presents the overall ESWO-based WSN deployment framework, including (1) the ESWO with ALS and DMPG, (2) sensor deployment and coverage evaluation, and (3) performance assessment in terms of coverage, convergence, obstacle resilience, and deployment reliability.

### 3.2. Enhanced Spider-Wasp Optimizer (ESWO)

ESWO builds on SWO [28] by adding an Adaptive Learning Strategy, dual-median-point guidance and a nest-overlap-avoidance update. Building on the approaches in [30,31], this learning scheme of “learning strategy” boosts population diversity by letting individuals learn from the differences between one another. The ESWO is improved by exploring the search space more effectively and avoiding premature convergence to local optima. The dual-median-point guidance strategy [32] is to lead the algorithm’s exploitation capabilities by guiding individuals towards promising regions of the search space based on midpoint of two randomly drawn elites.

#### 3.2.1. Adaptive Learning Strategy (ALS)

The learning strategy maintains a balanced exploration–exploitation trade-off by adaptively combining historical and current fitness information [30]. This symmetric learning mechanism promotes population diversity by utilizing information differences among individuals and reducing the risk of excessive exploration or exploitation. A learning factor (*LA*) and an experience factor (*EA*) are used to weigh the importance of the gaps that allow individuals to learn from diverse experiences and improve their search strategies. Four types of gaps are considered. At each iteration t, the population is ranked by fitness. The elite pool *E_top_* contains the top ⌈*ρN_p_*⌉ individuals, and the inferior pool *E_bot_* contains the remaining *N_p_*−⌈*ρN_p_*⌉ individuals. For each individual i, *X_better_* is drawn uniformly at random from *E_top_*\{*X_best_*, *X_i_*}, and *X_worse_* is drawn uniformly at random from *E_bot_*\ {*X_i_*}. Excluding *X_best_* prevents the degenerate case *S_t_*_1_ = 0. The four gaps are then: *S_t_*_1_, between the best individual and *X_better_*; *S_t_*_2_, between the best individual and *X_worse_*; *S_t_*_3_, between *X_better_* and *X_worse_*; and *S_t_*_4_, between two randomly selected individuals *X_a_* and *X_b_*(*a* ≠ *b* ≠ *i*). These gaps, denoted as *St_k_* (*k* = 1, 2, 3, 4), are mathematically defined as differences in their positions.

(9) *St*_1_ = ‖*X_best_* − *X_better_*‖_1_, *St*_2_ = ‖*X_best_* − *X_worse_*‖_1_,    *St*_3_ = ‖*X_better_* − *X_worse_*‖_1_, *St*_4_ = ‖*X_a_* − *X_b_*‖_1_

where ‖·‖_1_ is the L1 norm of the position difference. *X_better_* and *X_worse_* are drawn independently for each individual i and each iteration. If *E_top_* \{*X_best_*,*X_i_*} or *E_bot_* /{*X_i_*} is empty, which can occur only for *N_p_* < 4, the draw is made from the other pool; with *N_min_* = 10 and ρ = 0.5 this case does not arise. The gaps *S_t_*_1_, …, *S_t_*_4_ are scalars; they are used only through the normalized factor *EA_k_* in Equation (10). The learning factor and experience factor are $LA_{i}=\frac{F\left(X_{i}^{t}\right)}{F_{\max}^{t}}$, $EA_{k}=\frac{St_{k}}{\sum_{m=1}^{4}St_{m}}$, k = 1,…,4, with $F_{\max}^{t}=\max_{i}F\left(X_{i}^{t}\right)$. The ALS step is

(10) $$X_{i}^{\mathrm{new}}=X_{i}^{t}+\omega_{k^{*}}\;LA_{i}\;\left(X_{a}^{t}-X_{i}^{t}\right)+r_{4}\;EA_{k^{*}}\;\left(X_{\mathrm{best}}^{t}-X_{i}^{t}\right),\;\;k^{*}=\arg\max_{k}EA_{k},$$

where $X_{i}^{\mathrm{new}}$ is the updated position of wasp i, *r*_4_ ~ *U*(0,1), *ω_k_*∗ is the learning weight of the dominant gap type, and $X_{\mathrm{best}}^{t}$ is the current best individual. The learning process enhances the population’s diversity and expands the exploration of the search space, improving the algorithm’s ability to find the global optimum, factors weigh the importance of each gap, enhancing diversity and exploration.

#### 3.2.2. Dual-Median-Point Guidance Strategy (DMPG)

Effective exploitation requires guiding individuals toward promising regions while retaining population diversity. To this end, ESWO employs a dual-median-point guidance strategy (DMPG) that uses two elite individuals as guide points for each population member [32]. Unlike a population mean, which is identical for all individuals, DMPG samples two elite individuals separately for each (i), resulting in potentially different guide points across individuals. The two elites are averaged to give an individual guide point for each member. The dual-median-point guidance is defined as

(11) $$X_{\mathrm{guide},i}=1/2\left(X_{i_{1}}^{t}+X_{i_{2}}^{t}\right),$$

where *X_guide_*_,_*_i_* denotes the midpoint of two elite solutions associated with the *i*-th individual at iteration *t*. For each individual i, two distinct individuals $X_{i_{1}},X_{i_{2}}$ are drawn uniformly from the elite pool *E_top_* (top ⌈*ρN_p_*⌉ by fitness), with *i*_1_ ≠ *i*_2_ ≠ *i*. The bootstrapping distance is calculated as follows

(12) $$\gamma_{i}=X_{\mathrm{guide},i}-X_{i}^{t},\;X_{i}^{\mathrm{new}}=X_{i}^{t}+Q\left(t\right)\;r⊙\gamma_{i},$$

where *Q* is the guidance strength parameter that controls the influence of the dual-median guidance, r ∼ *U*(0,1)*^D^*, and ⊙ represents element-wise multiplication. The bootstrapping distance can be added as with expression.

(13) $$Q\left(t\right)=Q_{\min}+\left(Q_{0}-Q_{\min}\right)\left(1-\frac{t}{t_{\max}}\right),\;Q_{0}=0.25,\;Q_{\min}=0.05,$$

This mechanism enhances the algorithm’s exploitation capabilities. Furthermore, since different individuals possess distinct binary median points, the algorithm’s exploratory ability is preserved throughout the guidance process. Therefore, the introduction of the dual-median-point guidance strategy in the subsequent stages of the original SWO effectively ensures both rapid convergence and improved accuracy in solving the WSN coverage problem.

#### 3.2.3. Combined Update and Nest-Overlap Avoidance

The complete enhanced position update combines both learning and dual-median guidance strategies:

(14) $$X_{i}^{\mathrm{new}}=X_{i}^{t}+\omega_{k^{*}}LA_{i}\left(X_{a}^{t}-X_{i}^{t}\right)+r_{4}EA_{k^{*}}\left(X_{\mathrm{best}}^{t}-X_{i}^{t}\right)+Q\left(t\right)\;r⊙\gamma_{i},$$

where ω and *Q* are a weight factor and the guidance strength parameter, as mentioned in the previous equation; $X_{a}^{t}$ is the position of a randomly selected individual from the population at iteration (t). This thorough update approach balances exploration via diverse learning and exploitation through dual-median guidance, establishing a strong optimization framework. Avoiding overlapping nests is achieved using the following equation:

(15) $$X_{i}^{\mathrm{new}}=X_{a}^{t}+r\;\gamma_{i}⊙\left(X_{a}^{t}-X_{i}^{t}\right)+\left(1-r\right)\;U⊙\left(X_{b}^{t}-X_{c}^{t}\right),$$

with *a*, *b*, *c*, *i* pairwise distinct, *r* ∼ *U*(0,1), U ∼ *U*(0,1)*^D^*, and $X_{b}^{t}$, $X_{c}^{t}$ are randomly selected individuals from the population at iteration (*t*). The ESWO integrates the above the strategies into the original SWO framework. The learning strategy enhances population diversity during the searching phase. The dual-median-point guidance strategy refines exploitation during the following and escaping phases. This combined approach yields a more efficient optimization algorithm for maximizing WSN coverage under energy constraints. ESWO is expected to outperform SWO and similar methods in coverage, energy efficiency, and convergence speed. Performance details follow in the next subsection.

### 3.3. WSN Optimal Coverage with ESWO Algorithm

Sensor node coverage model is described how sensor coverage is modeled in a monitoring area [48]. A region *Ω* = [0,*W*] × [0,*L*] is monitored by *M_s_* homogeneous nodes with positions *s_a_* = (*p_a_*,*q_a_*), *a* = 1, …, *M_s_*. The decision vector is $X=\left(p_{1},q_{1},\ldots ,p_{M_{s}},q_{M_{s}}\right)\in R^{D}$ with *D* = 2*M_s_*, bounded by *L_j_* = 0 and *H_j_* = *W* or *L* [41]. Sensor nodes can update their positions over time without restrictions. Each node has a sensing radius *R_s_* and communication radius *R_c_* ≥ *R_s_*. The sensor node is assumed to be in the center of its sensing range with radius *R_S_*, *j* represents the target monitoring points in the 2D area: {*j*_1_, *j*_2_, *j*_3_, …, *j_m_*}. In each target point, *j**_b_*** is deployed in a position with coordinates (*p_b_*,*q_b_*). Real-world WSN deployment faces several practical challenges that impact sensor placement optimization. Terrain impact: Topographical features like hills, vegetation, and urban structures create irregular coverage patterns unlike idealized circular models [49]. Communication limitations result from atmospheric conditions, obstacles, and interference, which can reduce effective communication range and cause time-varying connectivity [50]. Energy limitations arise from the finite battery capacity of sensor nodes and the need for sustained operation with minimal maintenance [51]. Environmental conditions, including temperature, humidity, dust, and extreme weather, can further affect sensing accuracy and reliability [52]. Accordingly, ESWO favors deployment configurations that reduce exposure to adverse conditions while satisfying the required coverage and connectivity constraints.

Based on target monitoring points, a condition will be applied, i.e., if the distance between the target point, *jb* and any of the sensor node, *i_a_* is less than or equal to *RS* then this target point, *i_b_* is covered by that sensor node. Coverage determination is implemented with the target point coverage condition expressed as follows.

(16) $$\mathrm{cov}\left(a,j\right)=\left\{\begin{array}{ll} 1, & d\left(s_{a},g_{j}\right)\leq R_{s}\;\;\\ 0, & \mathrm{otherwise}, \end{array}\right.,$$

where *R_s_* is the sensing radius of the sensor node; *Cov (a*, *j)* is a binary variable. In coverage determination, sensing coverage depends on whether a target point lies within the sensing radius *R_s_* of at least one sensor node. Meanwhile, network connectivity relies on whether sensor nodes can communicate with neighboring nodes within the communication radius *R_c_*. A sensing radius defines an isotropic disk, so coverage is evaluated with the Euclidean distance:

(17) $$d\left(s_{a},g_{j}\right)=\sqrt{{\left(p_{a}-x_{j}\right)}^{2}+{\left(q_{a}-y_{j}\right)}^{2}}$$

Coverage is evaluated with this Euclidean distance throughout (binary-disk sensing model with radius *R_s_*); *d*(*s_a_*,*g_j_*) is the Euclidean distance between sensor node *a* at (*p_a_*,*q_a_*) and target point *g_j_* at (*x_j_*,*y_j_*). The energy-aware adjustment mechanism balances energy consumption across the network by considering both transmission power and sensing activity. The energy cost function for sensor node *i* is defined as:

(18) $$c_{j}\left(X\right)=1-\prod_{a=1}^{M_{s}}\left(1-\mathrm{cov}\left(a,j\right)\right)=\max_{a}\mathrm{cov}\left(a,j\right),\;C\left(X\right)=\frac{1}{M}\sum_{j=1}^{M}c_{j}\left(X\right)\in \left[0,1\right],$$

The continuous region Ω is discretized into a fixed set 𝒫 = { *g*_1_, …, *g_M_*} of M target points. Ω is divided into *n_x_* × *n_y_* equal cells of size Δ*_x_* = *W*/*n_x_* and Δ*_y_* = *L*/*n_y_*, and *g_j_* is the center of cell (u, v):

(19) *g_j_* = (*x_j_*, *y_j_*) = ((*u* − ½)Δ*_x_*, (*v* − ½)Δ*_y_*), *u* = 1, …, *n_x_*, *v* = 1, …, *n_y_*, *j* = (*v* − 1)*n_x_* + *u*,

with *M* = *n_x_n_y_* points and grid spacing Δ*_x_* = *W*/*n_x_*, Δ*_y_* = *L*/*n_y_*. The objective is the following fitness function:

(20) $$\max_{X\in {\left[L,H\right]}^{D}}\;F\left(X\right)=C\left(X\right)-\lambda_{E}\;\Phi_{E}\left(X\right)-\lambda_{R}\;\Phi_{R}\left(X\right),\;\;\lambda_{E}=0.1,\;\lambda_{R}=0.05,$$

where *Φ_E_*(*X*) and *Φ_R_*(*X*) are dimensionless penalty terms in [0,1] measuring violation of the energy constraints. The goal is to maximize *F(X)*, i.e., the covered fraction of target points minus the weighted energy-constraint violation.

(21) *X_best_* = argmax*_i_F*(*X_i_*),

Over the evaluated population, higher *F* is better. It is utilized within the ESWO optimization algorithm to determine the optimal configuration for target deploying network. This function integrates multiple aspects of WSN performance—primarily maximizing coverage—while incorporating energy consumption constraints. These constraints ensure that only feasible solutions that violate the energy constraints are penalized in the fitness. Algorithm 2 presents the pseudocode of the ESWO method designed to optimize the WSN nodes coverage.
**Algorithm 2:** ESWO for WSN Coverage
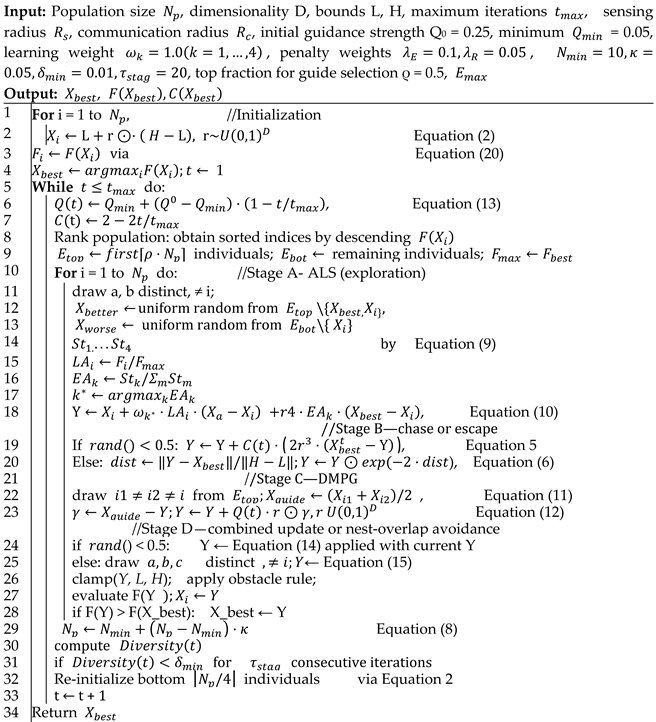


## 4. Simulation Results

This section presents a comprehensive evaluation of the ESWO algorithm for maximizing coverage efficiency in WSNs. ESWO’s performance is assessed through extensive simulations under varying network complexities, obstacle densities, and deployment conditions, and is compared with state-of-the-art optimization algorithms.

### 4.1. Experimental Settings

All simulations were implemented in MATLAB R2023b on an Intel Core i7-8250U workstation (8 cores, 1.60 GHz, 16 GB RAM). Four deployment scenarios of increasing scale were evaluated: (a) 25 nodes/50 × 50 m^2^; (b) 40 nodes/90 × 90 m^2^; (c) 50 nodes/100 × 100 m^2^; (d) 65 nodes/165 × 165 m^2^. Table 1 summarizes all parameter settings.

The simulation evaluation centered on three critical performance metrics: coverage ratio, convergence speed, and computational efficiency. Coverage ratio represents the percentage of the monitoring area effectively covered by the sensor network, with higher ratios reflecting superior sensing quality. Convergence speed is determined by the number of iterations needed to achieve a stable solution, where fewer iterations are advantageous for time-sensitive applications. Computational efficiency is assessed by the algorithm’s execution time, with lower overhead enabling real-time deployment in resource-limited settings. The experiments simulated realistic challenges in WSN deployment, incorporating obstacles such as buildings or mountains that create “monitoring blind regions” restricting sensor placement. These scenarios rigorously tested the algorithms’ ability to optimize coverage under practical constraints.

**Figure 3:**
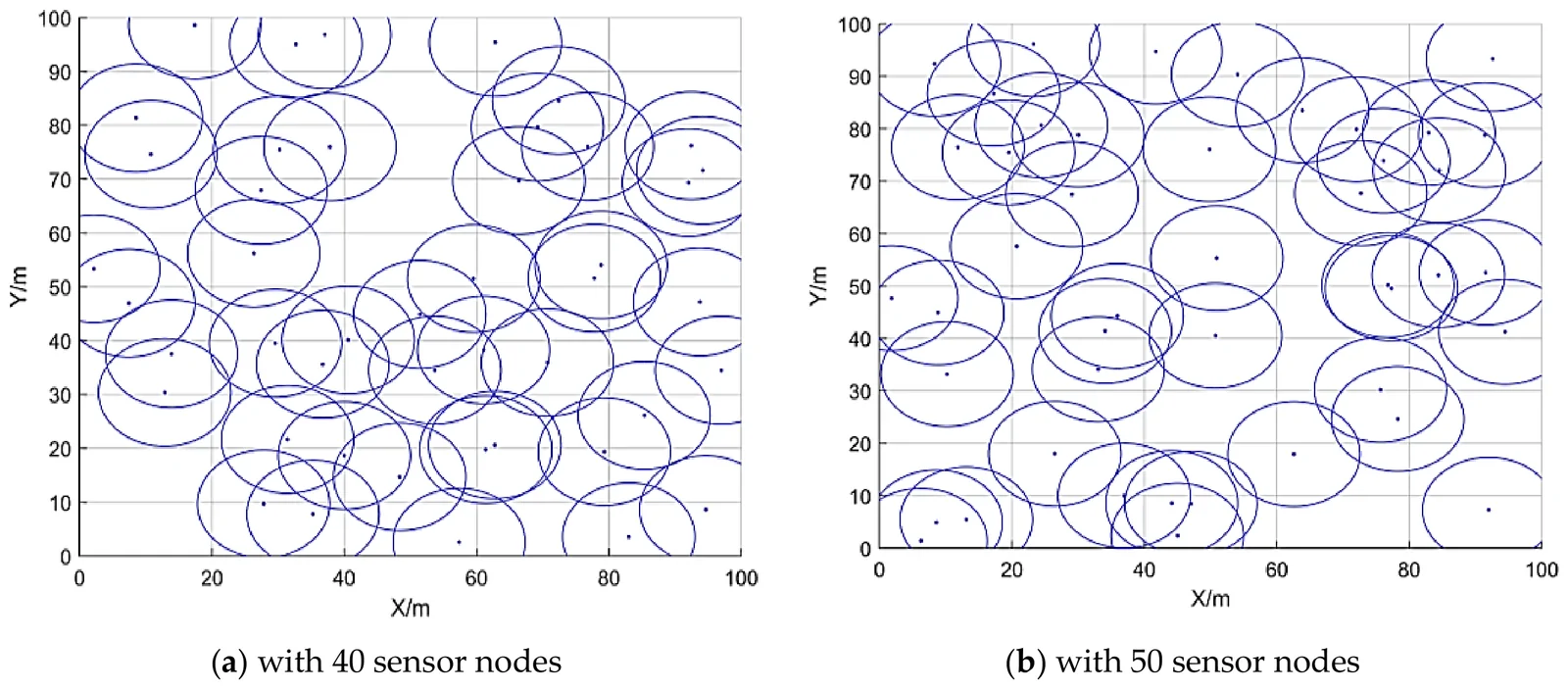
Graphical coverage diagrams illustrating the random initialization of sensor nodes used in the ESWO algorithm for coverage optimization: Initialization with (**a**) 40 sensor nodes and (**b**) 50 sensor nodes.

Figure 3 illustrates the random initial distribution of sensor nodes, represented through a graphical coverage diagram. This figure demonstrates the deployment of sensor nodes according to the ESWO algorithm, applied in a statistical coverage optimization scheme. The coverage patterns are shown for different sets of sensor nodes, with quantities of 50 and 65 nodes in area of 100 × 100 m^2^ respectively, highlighting the algorithm’s adaptability to varying node densities.

### 4.2. Coverage Performance and Convergence

To validate ESWO’s effectiveness, we compared its performance against seven state-of-the-art optimization algorithms: AGWO [40], ATSA [42], EPSO [41], EAOA [43], IHBA [45], MGWO [47], and the original SWO [28].

The parameters include population size: *N_P_*, maximum iterations: *t_max_*, boundaries: *H, L*, and specific parameters for each algorithm (e.g., random variables: *r*, transit probability: *p* learning rates: *l_r_*, weighting factors: *ω*), among other relevant settings. Table 2 provides an overview of the parameter settings utilized for various algorithms in this study. Each algorithm is accompanied by its corresponding parameters, which are critical for optimizing performance and achieving optimal outcomes.

Coverage Performance: To evaluate the performance of the proposed ESWO algorithm against the original SWO algorithm, we conducted a series of experiments focusing on convergence optimization of the algorithm’s processing behaviors. The experiments utilized a fitness function designed to maximize coverage under varying node densities and deployment areas.

The configurations allow for a comprehensive comparison of the algorithms’ effectiveness across different spatial and node density conditions.

**Figure 4:**
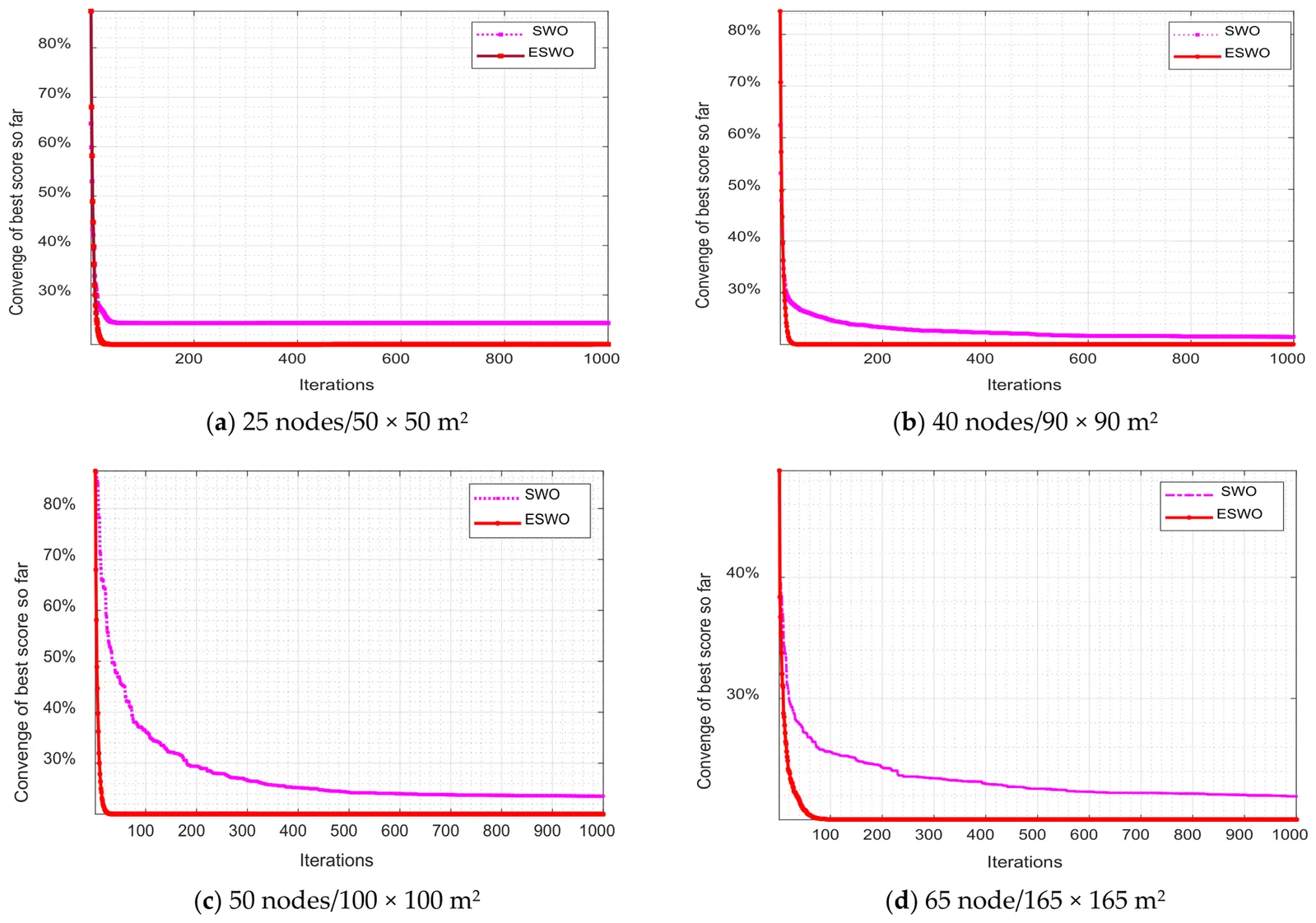
Coverage comparison between ESWO and SWO across four deployment scenarios: (**a**) 25 nodes in a 50 × 50 m^2^ area, (**b**) 40 nodes in a 90 × 90 m^2^ area, (**c**) 50 nodes in a 100 × 100 m^2^ area, and (**d**) 65 nodes in a 165 × 165 m^2^ area.

Figure 4 presents the results of this comparison, highlighting the converging performance of ESWO relative to SWO. The statistical convergence optimization scheme is evaluated across four deployment scenarios: (a) 25 nodes in a 50 × 50 m^2^ area, (b) 40 nodes in 90 × 90 m^2^, (c) 50 nodes in 100 × 100 m^2^, and (d) 65 nodes in 165 × 165 m^2^. The results illustrate that ESWO achieves improved coverage efficiency and reaches higher coverage than SWO in all four scenarios.

Table 3 presents a comparative analysis of coverage rates achieved by ESWO versus competing algorithms across different deployment scenarios.

A concise comparison of the performance of the proposed ESWO algorithm against existing techniques, including AGWO, ATSA, EAOA, EPSO, IHBA, MGWO, and SWO. The results, synthesized from experiments across various deployment scenarios, highlight ESWO’s superior coverage performance. Notably, in the 100 × 100 m^2^ deployment area, ESWO achieved an 92% coverage rate, outperforming the next best AGWO and EAOA 89%, while other algorithms ranged from 83% to 88%. To further assess the effectiveness of the proposed ESWO algorithm, we compared its performance with other existing approaches, including AGWO, ATSA, EAOA, EPSO, IHBA, MGWO, and SWO.

### 4.3. Convergence Speed Analysis

Convergence speed is a critical factor in the practical application of optimization algorithms, particularly for area coverage tasks. To evaluate this, we analyzed the number of iterations required by the proposed ESWO algorithm and other methods (AGWO, ATSA, EAOA, EPSO, IHBA, MGWO, and SWO) to achieve specific coverage levels in a 100 × 100 m^2^ deployment area with 50 nodes and a maximum of 1000 iterations. Table 4 reports the number of iterations required to achieve 70%, 75%, 80%, and 85% coverage in the 100 × 100 m^2^, 50-node scenario. It demonstrates ESWO’s faster convergence, attributed to its learning strategy and dual-median-point guidance, which efficiently directs the search toward high-quality solutions while maintaining population diversity.

ESWO achieves each milestone in the fewest iterations across all algorithms. The ALS and DMPG mechanisms collectively prevent the premature stagnation observed in SWO and enable ESWO to reach 85% coverage (523 iterations) substantially earlier than all competitors.

### 4.4. Coverage Error Rate and Deployment Visualization

To assess the accuracy of the proposed ESWO algorithm in optimizing node placement, we compared its coverage error rates against those of AGWO, ATSA, EAOA, EPSO, IHBA, MGWO, and SWO across multiple deployment scenarios.

**Figure 5:**
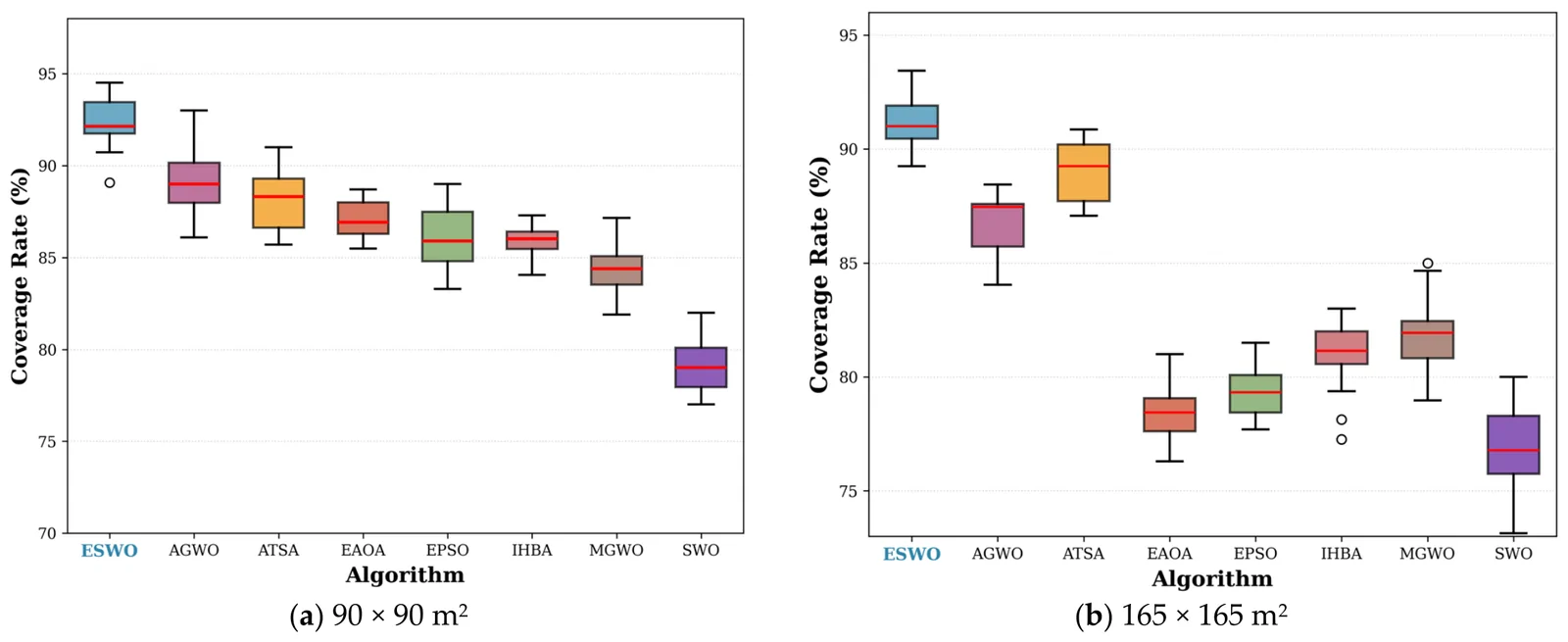
Boxplot comparison of the coverage performance distributions for ESWO, AGWO, ATSA, EAOA, EPSO, IHBA, MGWO, and SWO in 90 × 90 m^2^ and 165 × 165 m^2^ deployment scenarios.

Figure 5 presents boxplots illustrating the distribution of coverage performance for these algorithms in two deployment scenarios: 90 × 90 m^2^ and 165 × 165 m^2^. The boxplots highlight ESWO’s superior and more stable performance compared to the competing algorithms across these scenarios.

**Figure 6:**
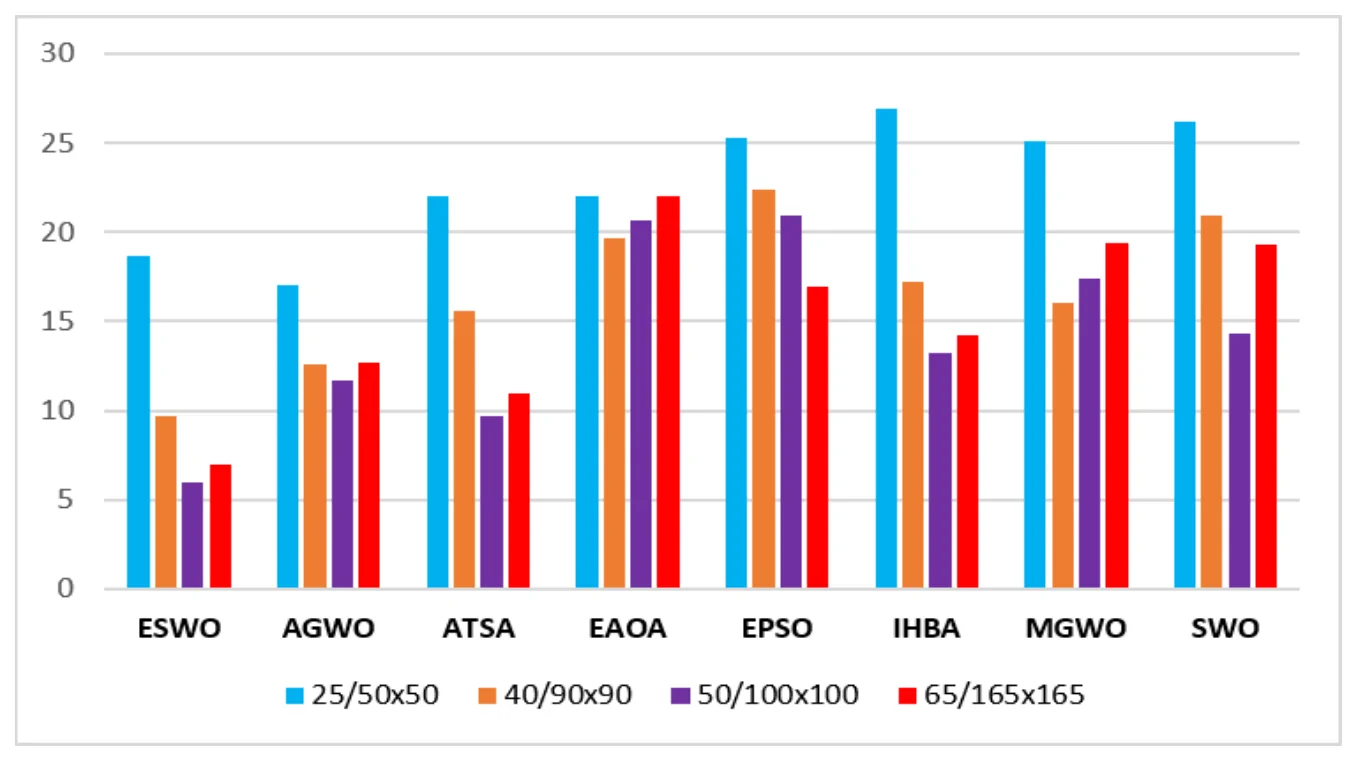
Bar chart comparing coverage error rates of ESWO, AGWO, ATSA, EAOA, EPSO, IHBA, MGWO, and SWO across four deployment areas: 50 × 50 m^2^, 90 × 90 m^2^, 100 × 100 m^2^, and 165 × 165 m^2^.

Figure 6 presents a bar chart illustrating the coverage error rates for these algorithms in four deployment areas: 50 × 50 m^2^, 90 × 90 m^2^, 100 × 100 m^2^, and 165 × 165 m^2^. The ESWO consistently achieves the lowest error rates across all scenarios, with its advantage becoming more pronounced in larger areas, owing to its effective balance between exploration and exploitation in the optimization process.

### 4.5. Robustness Under Varying Sensing Radii

**Figure 7:**
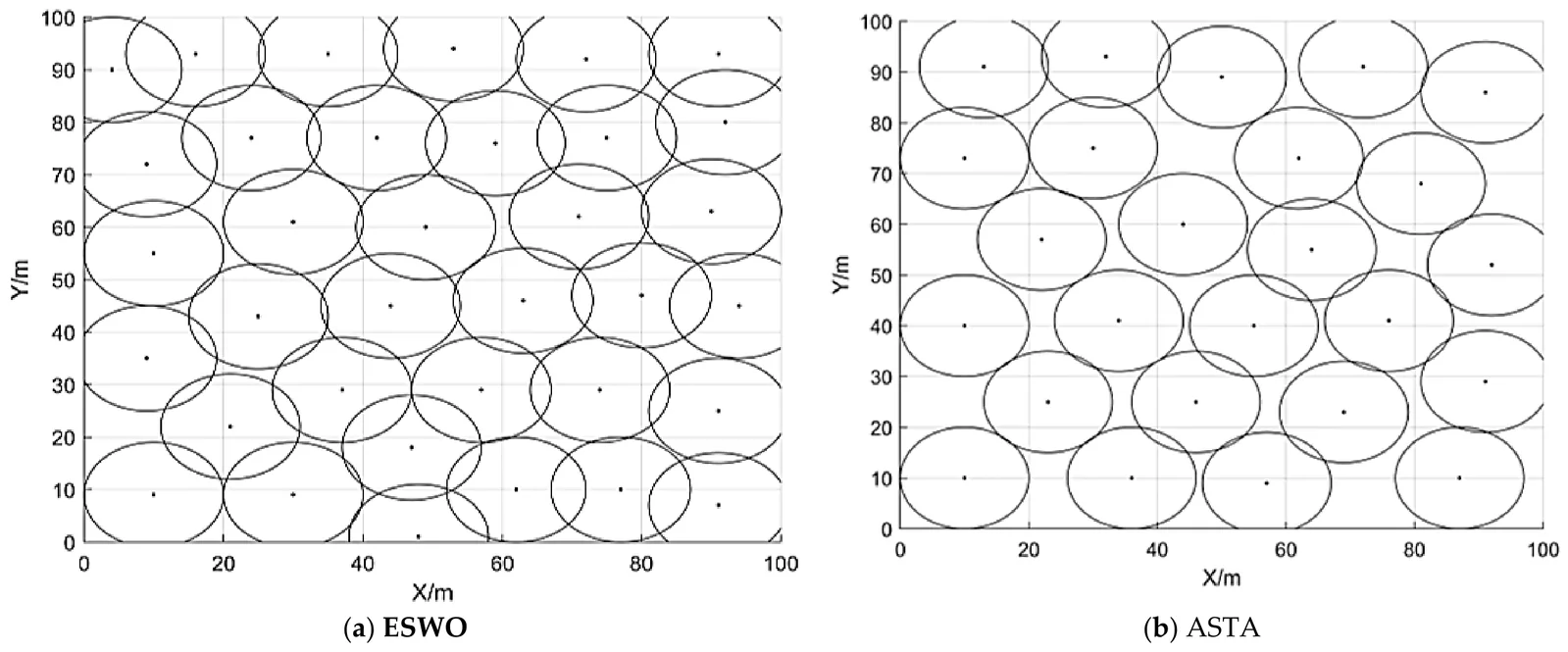

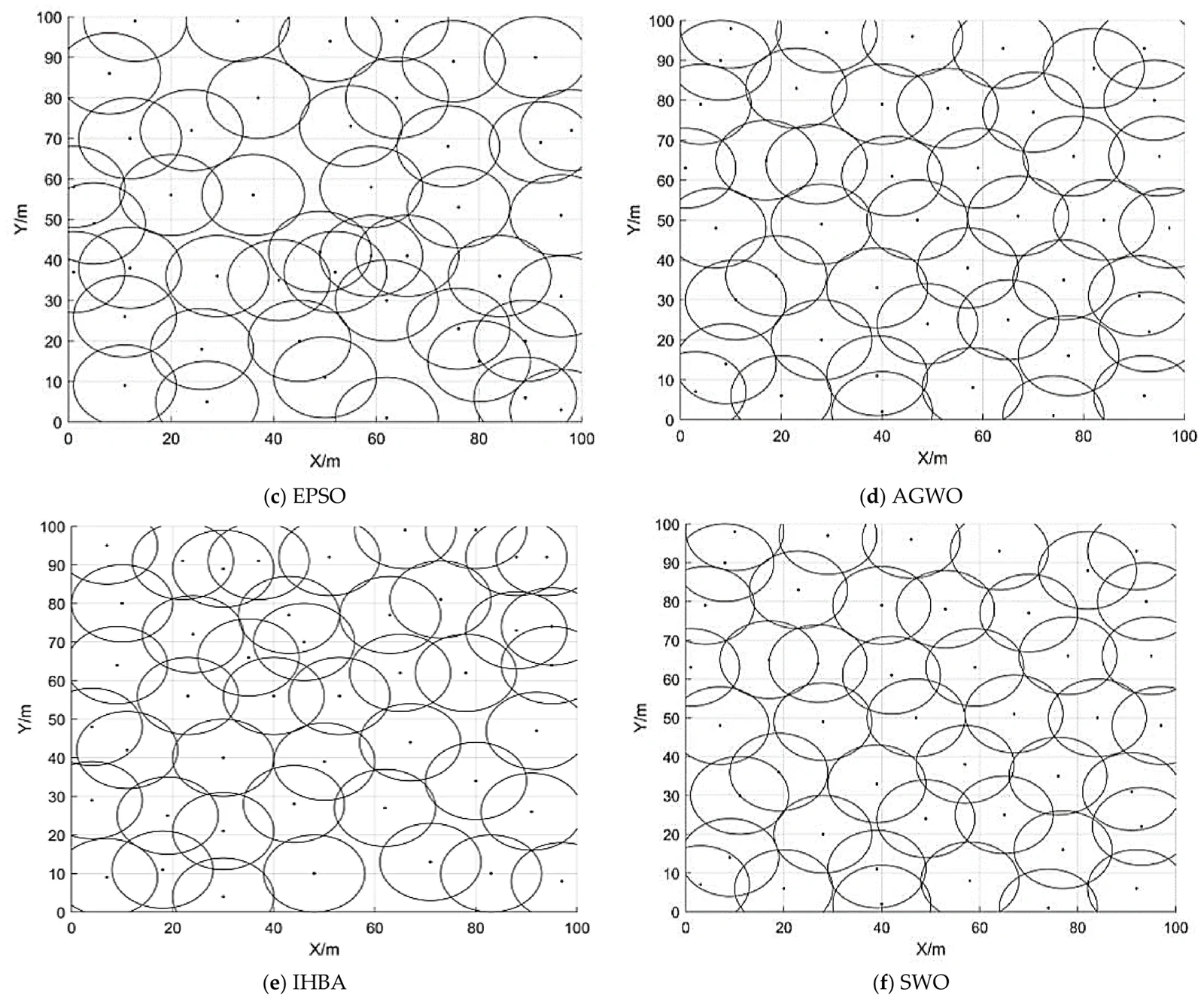
Graphical coverage comparison of node deployment patterns in a 100 × 100 m^2^ scenario for: (**a**) ESWO, (**b**) ATSA, (**c**) EPSO, (**d**) AGWO, (**e**) IHBA, and (**f**) SWO.

Coverage patterns were compared across different sensing radii to assess the robustness of the node placement strategies. Figure 7 illustrates the node deployment patterns generated by (a) ESWO, (b) ATSA, (c) EPSO, (d) AGWO, (e) IHBA, and (f) SWO in the 100 × 100 m^2^/50-node scenario. The sensing ranges are indicated by circles. ESWO achieves a more uniform node distribution with less coverage overlap, indicating improved coverage efficiency and resource utilization in the WSN.

**Figure 8:**
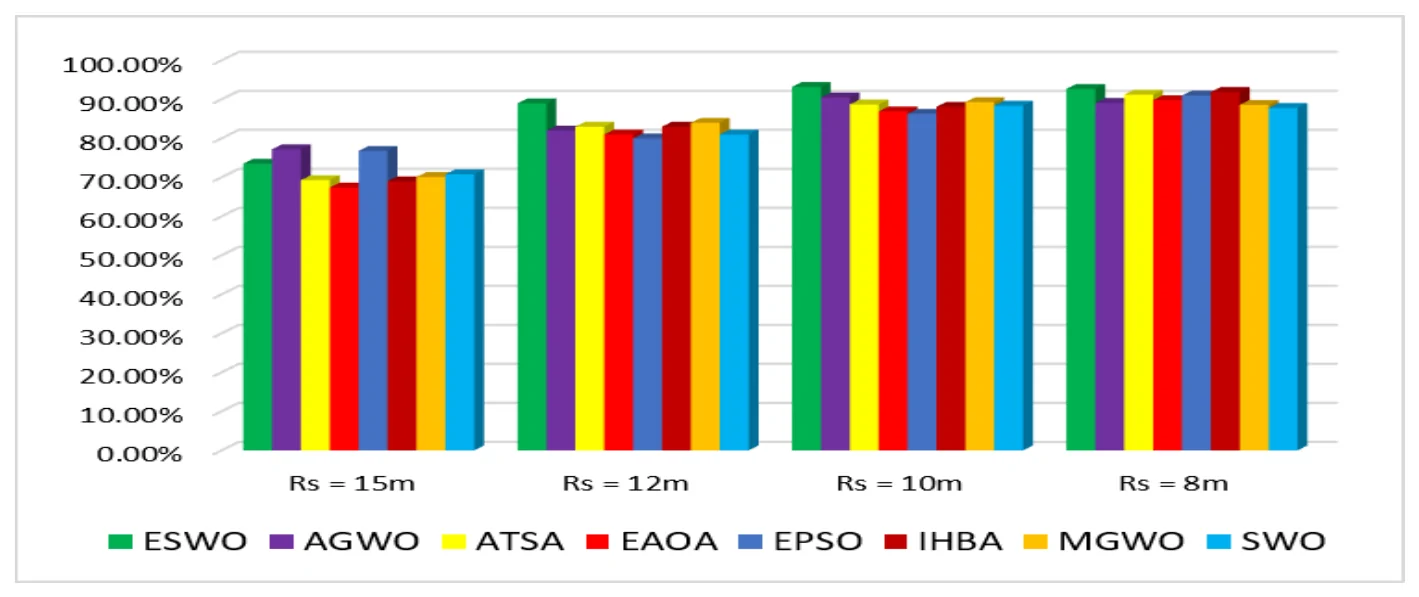
Performance comparison of ESWO and benchmark algorithms under varying sensing radii in a 100 × 100 m^2^ WSN with 50 nodes.

Circles represent sensing ranges, with ESWO demonstrating the most uniform distribution and minimal overlap. To evaluate the computational efficiency of the proposed ESWO algorithm compared to other metaheuristic algorithms (AGWO, ATSA, EAOA, EPSO, IHBA, MGWO, and SWO), we measured their time consumption across various deployment scenarios. The robustness of ESWO under varying sensing radii was evaluated in a 100 × 100 m^2^ deployment area with 50 nodes. Figure 8 compares ESWO with AGWO, ATSA, EAOA, EPSO, IHBA, MGWO, and SWO across different sensing-radius configurations. ESWO maintains stable coverage performance across the tested radii, demonstrating its robustness and adaptability to varying sensing constraints in WSNs.

### 4.6. Obstacle Adaptability

This subsection discusses the key findings from our experiments, focusing on the critical aspects: Energy Efficiency Analysis and Comprehensive Performance Evaluation. These analyses highlight the proposed ESWO algorithm’s effectiveness in optimizing coverage, convergence, execution time, and adaptability in WSN deployments.

To investigate the robustness of the proposed ESWO algorithm in realistic WSN scenarios, we evaluated its performance in environments with varying obstacle densities. Table 5 compares the coverage performance of ESWO against AGWO, ATSA, EAOA, EPSO, IHBA, MGWO, and SWO under these conditions. The results reveal that while all algorithms experience performance degradation with increasing obstacle density, ESWO consistently maintains a significant advantage, achieving 71.8% coverage even when 30% of the deployment area is obstructed, highlighting its adaptability to environmental constraints critical for real-world WSN deployments.

### 4.7. Energy Efficiency and Network Lifetime

Energy efficiency is paramount for battery-powered nodes in WSNs, as it directly impacts network lifetime. To evaluate the energy efficiency of the proposed ESWO algorithm, we analyzed its energy consumption based on node movement distances and estimated network lifetime compared to AGWO, ATSA, EAOA, EPSO, IHBA, MGWO, and SWO. Table 6 summarizes the results. ESWO achieves the shortest average node movement distance, indicating lower movement-related energy consumption under the same energy assumptions. This result may reflect ESWO’s ability to identify promising node positions with less movement; however, this explanation has not been independently verified.

**Figure 9:**
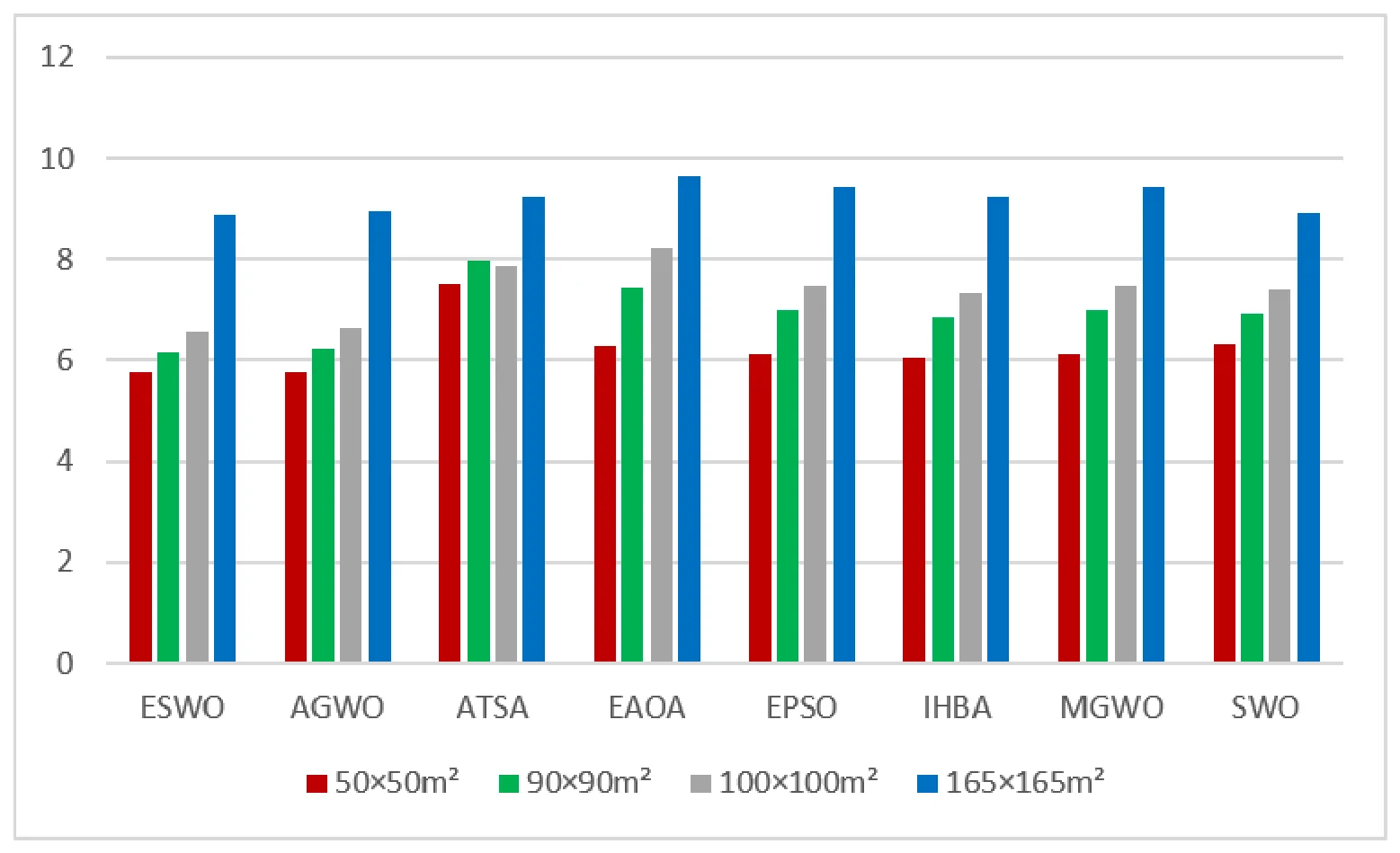
Bar chart comparing time consumption (in minutes) of ESWO, AGWO, ATSA, EAOA, EPSO, IHBA, MGWO, and SWO across four deployment areas: 50 × 50 m^2^, 90 × 90 m^2^, 100 × 100 m^2^, and 165 × 165 m^2^.

Table 6 reports a movement-based energy estimate. ESWO has the shortest average movement distance (18.7 m) and the lowest estimated energy (175.3 J). Figure 9 presents a bar chart comparing the time consumption in minutes for optimizing node placement in four deployment areas: 50 × 50 m^2^, 90 × 90 m^2^, 100 × 100 m^2^, and 165 × 165 m^2^. The results highlight ESWO’s ability to achieve efficient node placement with reduced computational time, particularly in larger deployment areas, underscoring its practical applicability in resource-constrained WSNs.

### 4.8. Statistical Reliability

To assess the statistical reliability of ESWO, 15 independent runs were conducted for each algorithm across multiple deployment scenarios. Table 7 summarizes the mean coverage, standard deviation, and success rate of ESWO, AGWO, ATSA, EAOA, EPSO, IHBA, MGWO, and SWO. ESWO achieves a mean coverage of 90.2%, a standard deviation of 1.2, and a success rate of 96%, indicating stable and reliable performance across repeated runs.

### 4.9. Effectiveness of the Algorithmic Enhancements

The effectiveness of the ESWO enhancements was evaluated in the largest deployment scenario (165 × 165 m^2^) using coverage, computational efficiency, and convergence metrics. Table 8 compares ESWO with the benchmark algorithms, demonstrating the combined effects of the proposed enhancements on coverage, efficiency, and convergence performance.

The overall effectiveness of ESWO was evaluated across multiple deployment scenarios using coverage rate, convergence, execution time, and obstacle adaptability. Table 9 summarizes the results against the benchmark algorithms, providing an integrated assessment of ESWO across key WSN coverage optimization metrics.

**Figure 10:**
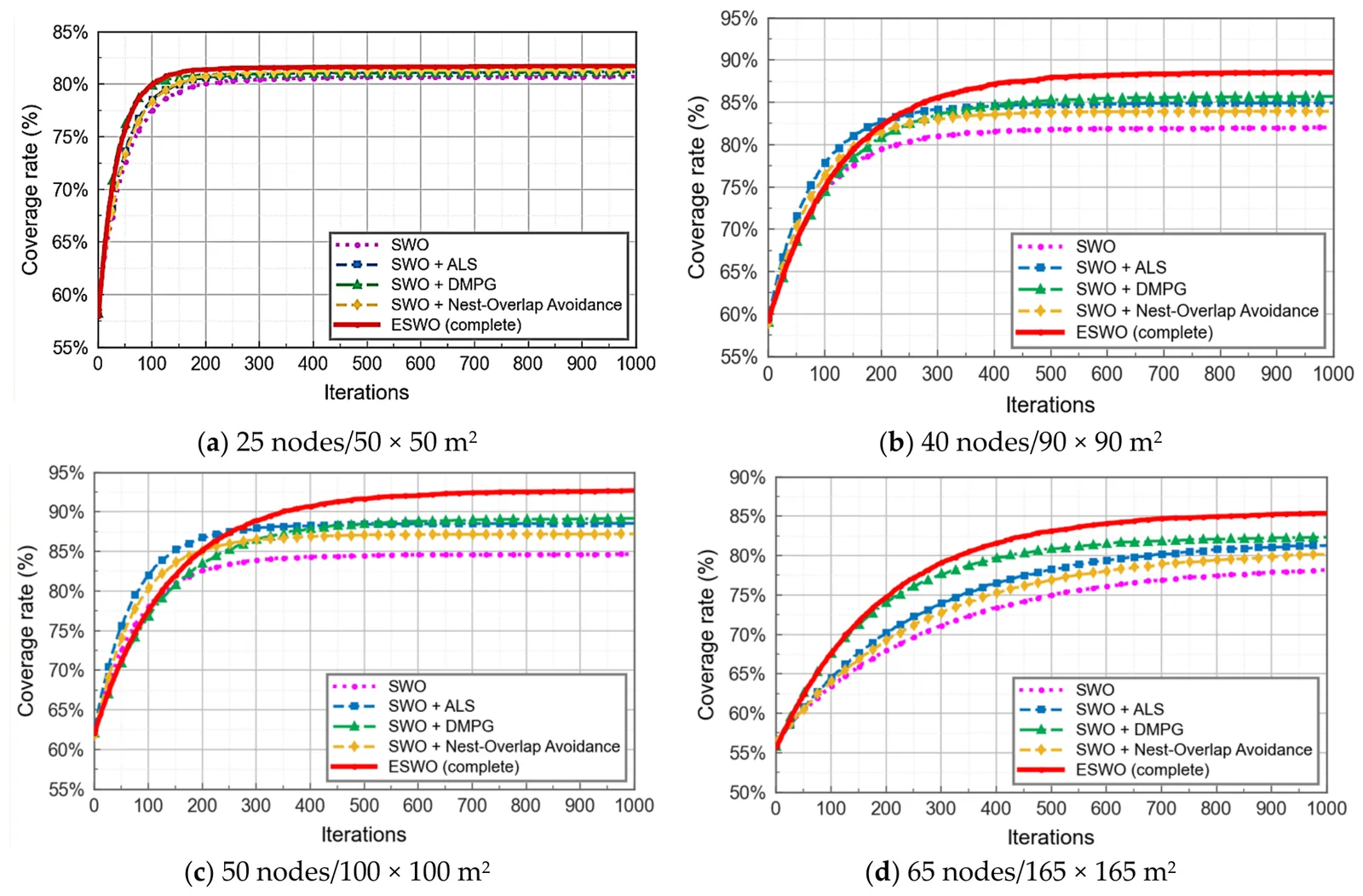
Coverage convergence comparison of ESWO and its ablation variants.

Figure 10 compares the coverage convergence of SWO, SWO + ALS, SWO + DMPG, SWO + NOA, and complete ESWO across four deployment scenarios: (a) 25 nodes in 50 × 50 m^2^, (b) 40 nodes in 90 × 90 m^2^, (c) 50 nodes in 100 × 100 m^2^, and (d) 65 nodes in 165 × 165 m^2^. Curves show the mean best-so-far coverage rate over 15 independent runs under identical experimental conditions.

### 4.10. Biomimetic Validity, Limitations, and Future Directions

The biomimetic basis of ESWO is grounded in the observable hunting and nesting behaviors of Pompilidae. The hunting cycle involves a transition from prey-searching to nesting, which is modeled in ALS through fitness-dependent exploration and exploitation. DMPG further reflects the preference for multiple high-quality reference points during nesting, reducing excessive convergence toward a single location.

Several limitations should be acknowledged. First, the experiments were conducted only in static, offline scenarios; performance under node mobility or failures remains unexplored. Second, the simplified energy model does not account for detailed path-loss effects or battery degradation. Third, the study considers only two-dimensional deployments, leaving three-dimensional and terrain-aware scenarios for future work. Fourth, the composite fitness function does not use formal Pareto-based multi-objective optimization, limiting detailed trade-off analysis. Finally, ESWO has not yet been validated on physical WSN hardware.

ESWO achieved the highest coverage in the reported scenarios; its convergence behavior was mixed (Table 3 and Table 4), and obstacle results are reported in Table 5. Real-world WSN deployments face several constraints not captured in current simulation environments. Future enhancements will address: (1) Integration with geographic information systems (GIS) for terrain-aware optimization incorporating digital elevation models and land cover data; (2) realistic propagation models accounting for path loss, shadowing, and interference effects; (3) dynamic adaptation mechanisms including fault detection and self-healing capabilities; and (4) scalability considerations for large-scale deployments with physical and economic constraints.

## 5. Conclusions

This paper presented the Enhanced Spider-Wasp Optimizer (ESWO) for WSN coverage optimization. ESWO extends SWO with an Adaptive Learning Strategy based on four positional gaps, a dual-median-point guidance mechanism based on the midpoint of two elite solutions, and a nest-overlap-avoidance update; the link to Pompilidae foraging and nesting is a conceptual rationale, not a biological validation. In the four simulated scenarios, ESWO gave the highest coverage among the eight algorithms (tied with MGWO at 50 × 50 m^2^) and reached the 70%, 75% and 85% thresholds in the 100 × 100 m^2^ scenario in the fewest iterations, although its final-convergence iteration counts were not uniformly the lowest. It also retained the highest coverage at 10–30% obstacle area and the lowest estimated energy consumption. ESWO may therefore be a practical option for WSN deployment planning when coverage is the primary objective and energy use is a constraint.

The findings are limited to static, two-dimensional simulations with a simplified energy model, and no component-wise ablation was performed, so the individual contributions of ALS, DMPG and nest-overlap avoidance are not isolated. Future work will add ablation studies, dynamic and terrain-aware deployments with realistic propagation models, multi-objective formulations, and tests on physical sensor hardware.

## Figures and Tables

**Table 1:** Simulation parameter settings for ESWO and WSN deployment.

Group	Parameter	Description	Value(s)
Scenario	W × L	Deployment area (m^2^)	50 × 50, 90 × 90, 100 × 100, 165 × 165
Scenario	*M_s_*	Number of sensor nodes	25, 40, 50, 65
Scenario	*R_s_*	Sensing radius (m)	8, 10, 12, 15
Scenario	*R_c_*	Communication radius (m)	20.03
Scenario	*n_x_* × *n_y_*, *M*	Target-grid cells; number of target points	2500; 8100; 10,000; 27,225
Scenario	*L_j_*, *H_j_*	Search bounds per dimension	0; W or L
Scenario	Obstacle area	Obstacle fraction (Table 5)	0, 10, 20, 30%
Common	*N_p_*	Population size, all algorithms	50
Common	*t_max_*	Maximum iterations, all algorithms	1000
Common	*Runs*	Independent runs per algorithm	[10 or 15]
SWO base	*p_th_*, *α_e_*	Probability threshold; decay rate	0.5, 2.0
ESWO	*N_min_*	Minimum population size	10
ESWO	*κ*	Population reduction factor	0.05
ESWO	*ρ*	Elite fraction for DMPG	0.5
ESWO	*Q*_0_/*Q_min_*	Initial/minimum guidance strength (linear decay)	0.25/0.05
ESWO	*ω_k_*	ALS learning weights	1.0 (k = 1…4)
ESWO	𝛿*_min_*/*τ_stag_*	Diversity threshold/stagnation window	0.01/20
ESWO	Restart fraction	Individuals re-initialized on stagnation	⌊*N_p_*/4⌋
ESWO	*λ_E_*, *λ_R_*	Fitness penalty weights	0.1, 0.05
ESWO	*E_max_*	Energy bound	*E_max_* = *e_m_* · *M_s_*·√(*W*^2^ + *L*^2^)

**Table 2:** Parameter settings for algorithms.

Algorithm	Parameter Settings	
	*Np* and *t_max_*	Other Parameters
AGWO	*Np* = 50, *t_max_* = 1000	*a ϵ* [2 *to* 0], *r*_1_, *r*_2_ *ϵ* [0,1], A = *2a·r*_1_ − a, *C = 2·r*_2_, α = 0.5, β = 0.8
ATSA	*Np* = 50, *t_max_* = 1000	*p* = 0.7, Step size = 0.1, *R* = 2.0, Exploration factor: *p*_1_ = 0.3, Exploitation factor *p*_2_ = 0.7
EPSO	*Np* = 50, *t_max_* = 1000,	*w ϵ* [0.9 *to* 0.4], *c*_1_, *c*_2_ = 2.0, *V_max_* = 0.2 · (*X_max_* − *X_min_*), Constriction factor α = 0.729
EAOA	*Np* = 50, *t_max_* = 1000,	Acceleration coefficient α = 2.0, Density factor *d* = 1000, V = 0.5, C_1_ = 2·*rand()*, C_2_ = 2·*rand()*, C_3_ = 2·rand()
IHBA	*Np* = 50, *t_max_* = 1000	β = 6, C = 2, Honey badger intensity *d* = 0.5, Digging capability *c_r_* = 0.8, Smelling capability = 0.3
MGWO	*Np* = 50, *_Tmax_* = 1000,	a = decreases linearly from 2 to 0, Strategy selection probability *p* = 0.3, Lévy flight parameter ***ε*** = 1.5, Crossover rate *c_r_* = 0.6
SWO	*Np* = 50, *t_max_* = 1000,	Probability threshold *p_th_* = 0.5, decay rate *α_e_* = 2.
ESWO	*Np* = 50, *t_max_* = 1000,	Guidance strength Q_0_ = 0.25, minimum *Q_min_* = 0.05, penalty weights *λ_E_* = 0.1, *λ_R_* = 0.05, *N_min_* = 10, *κ* = 0.05, 𝛿*_min_* = 0.01, *τ_stag_* = 20

**Table 3:** Performance comparison of ESWO with AGWO, ATSA, EAOA, EPSO, IHBA, MGWO, and SWO across various deployment scenarios.

Approach	Metric	50 × 50 m^2^	90 × 90 m^2^	100 × 100 m^2^	165 × 165 m^2^
ESWO	Coverage rate (%)	81	88	92	89
	Nodes deployed	25	40	50	65
	Convergence iterations	146	503	556	765
	Execution time (m)	5.75 × 10^0^	6.15 × 10^0^	6.57 × 10^0^	8.87 × 10^0^
AGWO	Coverage rate (%)	79	83	89	81
	Nodes deployed	25	40	50	65
	Convergence iterations	396	493	698	754
	Execution time (m)	5.78 × 10^0^	6.22 × 10^0^	6.65 × 10^0^	8.95 × 10^0^
ATSA	Coverage rate (%)	80	81	88	82
	Nodes deployed	25	40	50	65
	Convergence iterations	365	433	669	954
	Execution time (m)	7.52 × 10^0^	7.98 × 10^0^	7.86 × 10^0^	9.24 × 10^0^
EAOA	Coverage rate (%)	78	80	89	80
	Nodes deployed	25	40	50	65
	Convergence iterations	623	412	712	932
	Execution time (m)	6.26 × 10^0^	7.44 × 10^0^	8.23 × 10^0^	9.64 × 10^0^
EPSO	Coverage rate (%)	79	84	86	81
	Nodes deployed	25	40	50	65
	Convergence iterations	465	433	673	954
	Execution time (m)	6.12 × 10^0^	6.98 × 10^0^	7.46 × 10^0^	9.44 × 10^0^
IHBA	Coverage rate (%)	80	79	87	80
	Nodes deployed	25	40	50	65
	Convergence iterations	451	555	675	876
	Execution time (m)	6.06 × 10^0^	6.84 × 10^0^	7.31 × 10^0^	9.25 × 10^0^
MGWO	Coverage rate (%)	81	82	84	79
	Nodes deployed	25	40	50	65
	Convergence iterations	334	444	644	755
	Execution time (m)	6.42 × 10^0^	6.18 × 10^0^	7.56 × 10^0^	9.54 × 10^0^
SWO	Coverage rate (%)	80	81	82	78
	Nodes deployed	25	40	50	65
	Convergence iterations	145	511	645	761
	Execution time (m)	6.29 × 10^0^	6.91 × 10^0^	7.38 × 10^0^	10.23 × 10^0^

**Table 4:** Convergence analysis: iterations to specified coverage levels in 100 × 100 m^2^ with 50 nodes (*t_max_* = 1000).

Algorithm	Iterations to 70% Coverage	Iterations to 75% Coverage	Iterations to 80% Coverage	Iterations to 85% Coverage
ESWO	98	206	384	523
AGWO	104	235	398	682
ATSA	143	276	421	650
EAOA	167	295	456	698
EPSO	152	287	432	651
IHBA	128	249	387	634
MGWO	121	233	396	612
SWO	120	213	397	601

**Table 5:** Performance comparison of ESWO, AGWO, ATSA, EAOA, EPSO, IHBA, MGWO, and SWO in environments with varying obstacle densities.

Algorithm	No Obstacles	10% Obstacle Area	20% Obstacle Area	30% Obstacle Area
ESWO	92.0%	86.3%	80.5%	71.8%
AGWO	89.0%	78.6%	70.2%	61.4%
ATSA	88.0%	79.8%	72.3%	63.5%
EAOA	81.0%	77.4%	68.9%	60.2%
EPSO	80.0%	76.5%	68.1%	59.7%
IHBA	83.5%	79.3%	72.1%	62.9%
MGWO	84.0%	80.1%	73.3%	63.7%
SWO	81.0%	77.2%	69.8%	60.5%

**Table 6:** Comparison of node movement distances and estimated movement-related energy consumption for ESWO, AGWO, ATSA, EAOA, EPSO, IHBA, MGWO, and SWO.

Algorithm	Avg. Node Movement Distance (m)	Estimated Energy Consumption (J)	Estimated Network Lifetime (h)
ESWO	18.7	175.3	241.5
AGWO	19.4	214.6	198.7
ATSA	19.6	203.1	201.3
EAOA	19.9	218.7	194.3
EPSO	19.1	219.8	185.9
IHBA	19.5	208.4	204.9
MGWO	20.01	200.5	202.4
SWO	19.3	196.8	207.2

**Table 7:** Statistical analysis of performance for ESWO, AGWO, ATSA, EAOA, EPSO, IHBA, MGWO, and SWO across 15 independent runs, based on mean coverage, standard deviation, and success rate.

Algorithm	Mean Coverage (%)	Standard Deviation	Best Run	Worst Run	Max Success Rate (%)
ESWO	90.2	1.2	96.7	86.3	95
AGWO	88.5	1.8	83.1	79.2	92
ATSA	87.3	1.5	86.0	80.1	93
EAOA	82.8	2.2	83.5	75.0	90
EPSO	85.4	1.9	82.3	78.1	91
IHBA	82.9	1.7	85.2	74.4	93
MGWO	84.6	1.6	85.9	76.8	92
SWO	81.9	1.7	79.8	76.5	91

**Table 8:** Performance comparison of ESWO and benchmark algorithms in a 165 × 165 m^2^ WSN deployment.

Approach	Coverage Rate (%)	Running Time (Minutes)	Convergence (%)	Monitoring Area
ESWO	88	8.87	94	165 m × 165 m
AGWO	81	8.95	92	165 m × 165 m
ATSA	82	9.24	92	165 m × 165 m
EAOA	80	9.65	90	165 m × 165 m
EPSO	81	9.44	91	165 m × 165 m
IHBA	80	9.25	93	165 m × 165 m
MGWO	79	9.44	93	165 m × 165 m
SWO	79	10.23	90	165 m × 165 m

**Table 9:** Overall performance comparison of ESWO and benchmark algorithms across key WSN coverage metrics.

Algorithm	Average Coverage (%)	Average Convergence Iterations	Running Time	Energy Consumption (J)	Coverage Retention at 30% Obstacles (%)
ESWO	92	556	6.57	175.3	80.7
AGWO	88	343	6.65	214.6	74.9
ATSA	87	563	7.86	203.1	76.5
EPSO	88	563	7.46	229.8	74.6
EAOA	85	623	8.23	218.7	74.3
IHBA	86	675	7.31	208.4	75.3
MGWO	85	544	7.46	200.5	75.8
SWO	86	349	7.38	196.8	74.7

## Data Availability

All data generated and analyzed in this study were obtained through simulation and are available from the corresponding author upon reasonable request.

## Abbreviations

The following abbreviations are used in this manuscript.

WSN	Wireless sensor network
ESWO	Enhanced Spider-Wasp Optimizer
SWO	Spider-Wasp Optimizer
ALS	Adaptive Learning Strategy
DMPG	Dual-Median-Point Guidance
AGWO	Aware Gray Wolf Optimizer
ATSA	Adapted Transit Search Algorithm
EPSO	Enhanced Particle Swarm Optimization
EAOA	Enhanced Archimedes Optimization Algorithm
IHBA	Improved Honey Badger Algorithm
MGWO	Multi-strategy Enhanced Gray Wolf Optimizer
Symbol	Meaning
*N_p_*, *N*_min_	Population size; minimum population size
*κ*	Population reduction factor
*t*, *t*_max_	Current iteration; maximum number of iterations
*D*	Dimension of the decision vector (*D* = 2*M_s_*)
*L*, *H*	Lower- and upper-bound vectors of the search space
*X*	Position (decision) vector of an individual
*M_s_*	Number of sensor nodes
*R_s_*, *R_c_*	Sensing radius; communication radius
*F*, *C*	Fitness value; coverage rate
*λ_E_*, *λ_R_*	Penalty weights
*Φ_E_*, *Φ_R_*	Dimensionless penalty terms in [0,1]
*Q*_0_, *Q*_min_	Initial and minimum guidance strength (DMPG)
*ρ*	Fraction of the population treated as elite for DMPG
*ω_k_*	ALS learning weights
𝛿_min_, *τ_stag_*	Stagnation threshold and stagnation length of the restart rule
